# Controlled Hypotension for Functional Endoscopic Sinus Surgery With Two Different Doses of Fentanyl

**DOI:** 10.7759/cureus.33859

**Published:** 2023-01-17

**Authors:** Pooja Giriyapur, Ravi Madhusudhana

**Affiliations:** 1 Anaesthesiology, Sri Devaraj Urs Medical College, Kolar, IND

**Keywords:** fentanyl, fess, general anesthesia, hemodynamic response, post-operative pain

## Abstract

Background and Objectives

Functional endoscopic sinus surgery (FESS) is a type of minimally invasive surgery done for acute and chronic sinus diseases or paranasal illnesses. The idea of FESS is to preserve the normal anatomy, which is non-obstructing and mucous membrane while removing tissue obstructing OMC (osteo metal complex) and facilitating drainage. The critical structures, including the brain, orbit, and carotid veins, the lack of adequate operating room, and bleeding that obscures endoscopic vision throughout the procedure may increase the likelihood of unfavorable surgical results. This study seeks to examine the hemodynamic effects of intubation and extubation as well as the impact of fentanyl infusion on lowering blood pressure during FESS procedures.

Materials and Methods

Sixty-eight patients from the American Society of Anesthesiologists classes 1 and 2 who were planned for functional endoscopic sinus operations were randomly split into two groups for this randomized prospective trial. Group 1 patient belonging to the fentanyl 2 mcg per kg bolus 30 minutes before induction followed by 2 mcg per kg per hr infusion for 90 minutes of surgery, and Group 2 patient belonging to fentanyl 1 mcg per kg bolus 30 minutes before induction followed by 1 mcg per kg per hr infusion for 90 minutes of surgery. The significance of the difference in quantitative measures was measured using the student-t test, and the Chi-square test was used to measure up the difference in proportion. Statistically significant was set at P<0.05.

Results

Mean systolic blood pressure was higher in members of Group 2 than in Group 1. In contrast to Group 2, Group 1 had considerably better surgical field conditions, surgeon satisfaction on the AONO'S scale, post-operative nausea and vomiting, and a post-operative VAS Score during the first 24 hours.

Conclusion

Pre-induction Fentanyl with infusion can effectively control hypotension during functional endoscopic sinus surgery.

## Introduction

The common procedure performed for paranasal sinus illness is FESS. The closeness of important structures, such as orbit, brain and carotid vessels, the lack of available operating room, and bleeding that obstructs endoscopic vision during the procedure may increase the likelihood of unfavourable surgical outcomes (like optic nerve trauma, dural puncture, hemorrhage, etc.) [1]. Due to these reasons, using induced hypotension during surgery is very vital [1].

A minimally invasive practice called functional endoscopic sinus surgery is used to treat chronic sinusitis. Small bleeding regions can make an operation harder to see and cause the spread of nearby structures. Using a variety of pharmacological drugs during general anesthesia, deliberate hypotension that is reducing the mean arterial pressure between 50 and 65 mmHg in normal blood pressure patients minimizes blood loss in various operations [2-7]. However, often prescribed hypotensive drugs can have unpleasant side effects, including heart blocks, rebound hypertension, tachyphylaxis, drowsiness, delayed recovery, and vasodilatation (halogenated substances, nitrates, and beta blockers, among others) [8-11]. These drugs are employed to provide a surgical field free of blood by achieving controlled hypotension [12-15].

This study is being proposed to find out the impact of predominant sympathoadrenal suppression, effects that is lower, heart rate and blood pressure of two different doses (1mcg per kg and 2mcg per kg) in facilitating controlled hypotension during functional endoscopic sinus surgery (primary objective). It is also proposed that this study can help control hypotension for patients undergoing FESS, which has not been done before in the institution.

## Materials and methods

Data source

We conducted a randomized control study in a tertiary care hospital in Kolar, Karnataka. The study involved 68 patients who were admitted for FESS under GA (general anesthesia) at R L Jalappa Hospital, Anaesthesiology Department, Sri Devaraj Urs Medical College, a constituent unit of Sri Devaraj Urs Academy of Higher Education and Research (SDUAHER), Tamaka, Kolar, during the academic year January 2021-June 2022. Based on Prabhat et al. study, the sample size was determined [1]. The mean heart rate at 5 min post-intubation was used as prevalence. Group A=85.72±15.28, Group B=77.45±14.30 was taken into consideration. Zα=1.96 at 95% CI, Z1-β=0.84 for 80% power of the study, assuming 10% of obsolete precision value. Total sample size = 68 out of that 34 in each group will be taken.

Ethical clearance

We did this study after getting ethical approval from the institutional ethics committee (SDUMC/KLR/IEC/614/2020-21).

Eligibility criteria and sampling

We included patients with age groups between 20 and 60 years, either male or female, American Society of Anesthesiologists status I and II, and patients undergoing FESS for sinusitis of nonfungal origin under GA. Exclusion criteria had the following conditions:

1) Patients with uncontrolled hypertension, uncontrolled diabetes mellitus, hyperresponsive airway disease from a previous illness, severe asthma, and known opioid hypersensitivity and allergy. 2) Patients with hepatorenal dysfunction, uncontrolled cardio-vascular illness, and severe respiratory and endocrine illness. 3) Patients with chronic smokers, alcohol abuse, substance abuse, and psychiatric disorders.

Data collection

Ethical clearance was obtained before starting the study. A thorough pre-anesthetic check-up was carried out, a history was taken, and a systemic examination was done. Vitals were noted, including the weight of the patient. Investigations asked before surgery were a complete hemogram, serum electrolytes, renal function test, bleeding time (BT) and clotting time (CT), ECG, and Chest X-ray. All patients were examined a day before the surgery, investigation reports were checked, the anesthetic procedure was explained, and informed consent was taken. Fasting was ensured for eight hours, and pre-medication for patients included tablets of 0.5 mg alprazolam and 150 mg ranitidine, which was repeated the morning of surgery. Patients were divided into two groups randomly.

Preparation of drug for infusion: Fentanyl 2ml containing 100µg was diluted with normal saline till 20cc so that the solution contained 5µg per ml. The drugs were administered using a syringe pump.

Patients were randomly divided into two groups: Group 1: Patient belonging to the fentanyl 2mcg/kg bolus 30 minutes before induction, followed by 2mcg/kg/hr infusion for 90 minutes of surgery. Group 2: Patient belonging to the fentanyl 1mg/kg bolus 30 minutes before induction followed by 1mcg/kg/hr infusion for 90 minutes of surgery. The infusion was stopped after 90 minutes intra-operatively. Parameters observed were surgical field condition, surgeon satisfaction profile, Aono's four-point scale, visual analog scale (VAS) score, postoperative nausea and vomiting score, and Ramsay sedation scale.

Statistical analysis

Data were gathered, coded, and entered into a Microsoft Excel spreadsheet (Redmond, USA). All the quantitative measures like heart rate, systolic blood pressure, and diastolic blood pressure were presented by mean confidence intervals, qualitative measures like Gender, and the American Society of Anesthesiologist physical status by proportions and confidence interval. The student-t test was used to decide the impact of the difference in the quantitative measurements. The proportional difference was compared using the Chi-square test. Statistics were considered significant if P <0.05.

## Results

**Figure 1:**
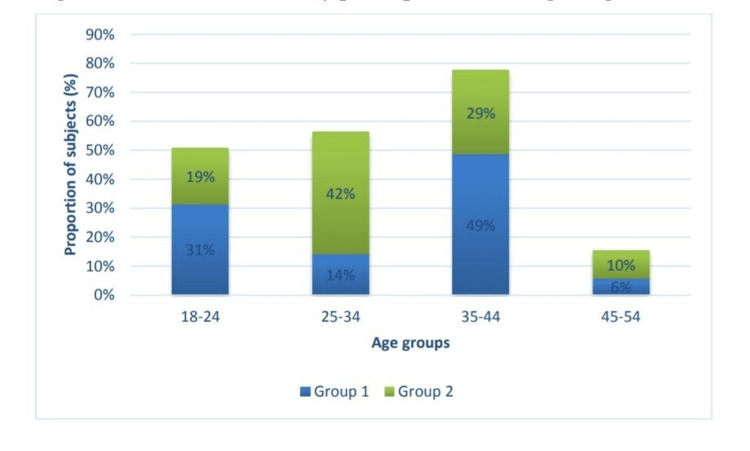
Distribution of study participants according to age

**Figure 2:**
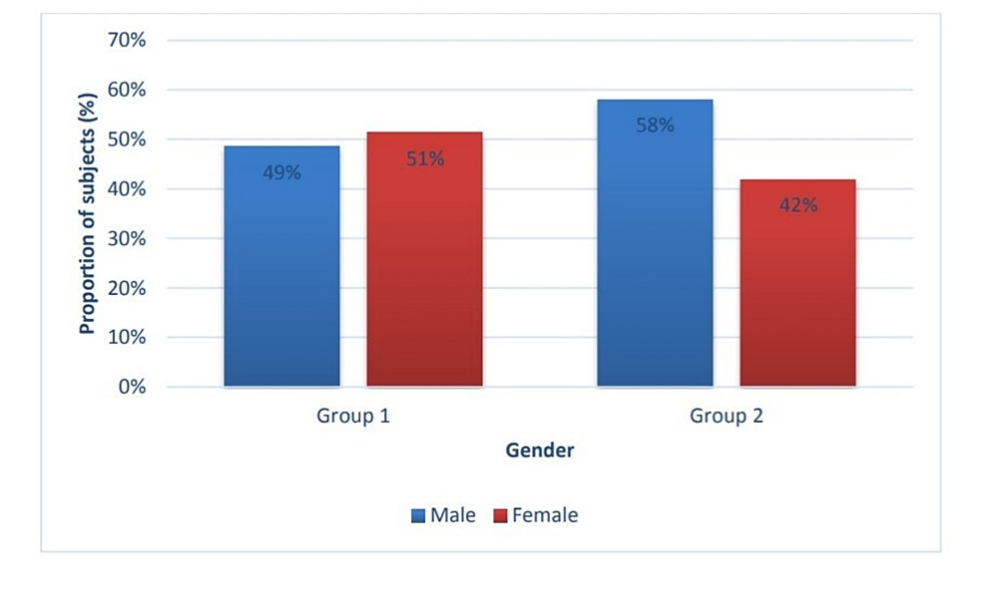
Distribution of study participants according to gender

**Figure 3:**
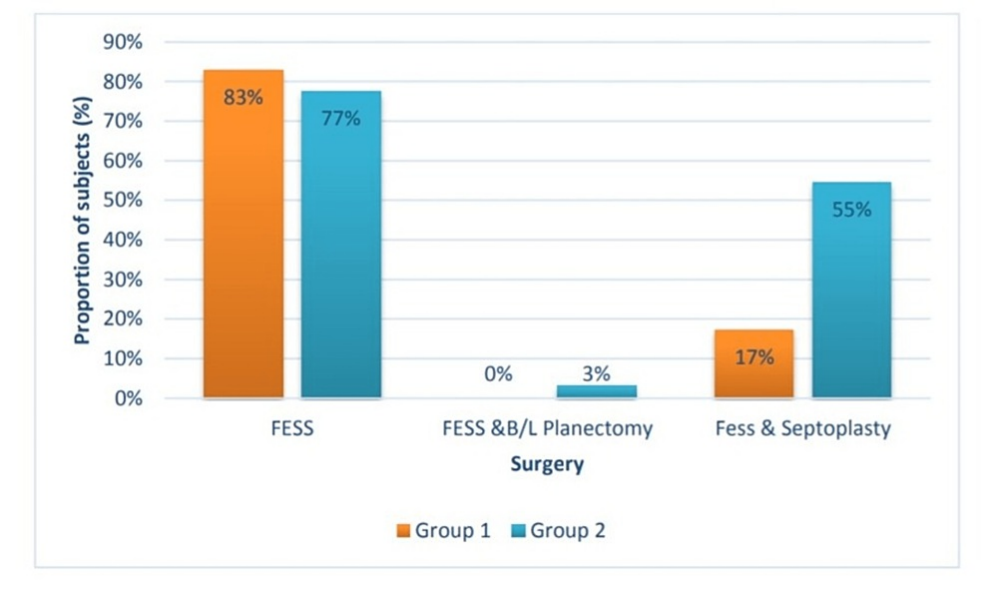
Distribution of study participants according to surgery

This study observed that most of the patients belonged to 35-44 the year's age group (Figure 1). Most patients were females (51.4%) in group 1 and males(58.1%) in group 2 (Figure 2), and most patients underwent FESS in group 1, and 17.2% had undergone both FESS and septoplasty. In group 2, 77.4% underwent FESS (Figure 3).

The mean heart rate was raised [80.71 (10.99)] among group 2 members more than the group 1 member [68.94 (8.11)] after 30 minutes of intubation per minute from the mean baseline heart rate. The statistical significance of this difference was high (p-value <0.001). Likewise, the two groups had significant associations after 45 minutes, 60 minutes, 75, and 90 minutes of intubation (p < 0.05).

In Table 1, the mean SBP was raised [138.1 (10.59)] among group 2 members than the group 1 member [124.3 (15.88)] after 1 minute of intubation. There was statistical significance (p-value < 0.05). Similarly, the two groups had a significant association after 5 minutes of extubation (p < 0.001).

**Table 1:** Comparison of systolic blood pressure among the patients during the treatment

Systolic Blood Pressure	Group 1	Group 2	P-value
	Mean, SD	Mean, SD	
Baseline	129.4, 12.17	128.5, 12.98	0.459
After Infusion	125.4, 12.55	127, 13.5	0.399
1 minute after intubation	124.3, 15.88	138.1, 10.59	0.042
3 minutes after intubation	120.1, 15.92	136, 14.01	0.3
5 minutes after intubation	115.1, 16.01	132.4, 15	0.249
10 minutes after intubation	111.7, 14.95	131.7, 10.1	0.004
15 minutes after intubation	109.5, 14.6	130.8, 10.53	0.004
30 minutes after intubation	108, 14.11	129.3, 10.74	0.027
45 minutes after intubation	108.4, 13.17	126.1, 12.55	0.425
60 minutes after intubation	107.2, 12.25	125.9, 11.33	0.726
75 minutes after intubation	106.2, 11.72	125.5, 11.09	0.821
90 minutes after intubation	107, 11.83	128.5, 12.61	0.965
1 minute after extubation	112.1, 14.62	138.2, 10.61	0.087
3 minutes after extubation	110.5, 14.04	137.3, 8.52	0.009
5 minutes after extubation	107.6, 13.62	134.1, 8.86	0.036
15 minutes after extubation	106.4, 11.64	129.9, 8.02	0.073
30 minutes after extubation	106.4,11.64	129.9, 8.02	0.084

The mean DBP was raised [84.09 (7.36)] among group 2 members than the group 1 member [68.4 (10.9)] after 10 minutes of intubation. Showed statistical significance here (p-value < 0.05). In Table 2, the MAP was raised [99.98 (7.56)] among group 2 members more than the group 1 member [82.87 (11.85)] after 10 minutes of intubation. Showed statistical significance (p-value < 0.05). Likewise, the two groups had a significant association after 15 minutes of intubation (p < 0.05). The MAP is raised [103.7 (8.56)] among group 2 members than the group 1 member [83.23 (12.66)] after 1 minute of extubation. Statistically significant was this difference (p-value < 0.01).

**Table 2:** Comparison of mean arterial blood pressure among the patients during the treatment

Mean Arterial Blood Pressure	Group 1	Group 2	P-value
	Mean, SD	Mean, SD	
Baseline	98.62, 8.13	97.78, 9.36	0.09
After Infusion	94.65, 8.44	96.19, 9.7	0.06
1 minute after intubation	94.23, 12.23	106.5, 9.31	0.247
3 minutes after intubation	90.2, 12.84	104.4, 11.2	0.31
5 minutes after intubation	85.59, 12.72	100.9, 11.89	0.26
10 minutes after intubation	82.87, 11.85	99.98, 7.56	0.003
15 minutes after intubation	80.52, 11.44	97.62, 8.02	0.026
30 minutes after intubation	78.92, 10.92	96.13, 8.52	0.15
45 minutes after intubation	79.05, 10.11	93.43, 9.04	0.601
60 minutes after intubation	78.3, 9.2	93.62, 9.04	0.798
75 minutes after intubation	77.32, 8.86	92.63, 9.31	0.864
90 minutes after intubation	77.92, 9.13	94.98, 10.61	0.583
1 minute after extubation	83.23, 12.66	103.7, 8.56	0.01
3 minutes after extubation	81.32, 11.82	102.6, 8.18	0.029
5 minutes after extubation	79.66, 10.93	100, 8.37	0.074
15 minutes after extubation	78.33, 9.46	96.82, 7.23	0.063
30 minutes after extubation	78.9, 8.81	94.34, 7.57	0.356

VAS score (Table 3), surgical field condition (Table 4), surgeon satisfaction score (Table 5), and Aono's four-point scale were superior in group 1 compared to group 2. According to the findings of our investigation, controlled hypotension could be started in all participants and maintained effectively. Patients who took fentanyl 2mcg/kg pre-induction showed improved surgical field conditions (SFC) and surgeon satisfaction scores (SSS). Patients who received a 2mcg/kg fentanyl infusion during FESS had better endoscopic images.

**Table 3:** Comparison of VAS score among the patients during the treatment

VAS Score	Group 1	Group 2	P-value
	Mean, SD	Mean, SD	
Baseline	1.57, 0.65	2.61, 0.49	0.033
6 hours	3.31, 0.79	4.96, 0.31	0.001

**Table 4:** Comparison of surgical field condition among the patients during the treatment

Surgical field condition	Group 1	Group 2
	n (%)	n (%)
No bleeding	0 (0)	0 (0)
Slight bleeding-no suctioning required	0 (0)	3 (9.7)
Slight bleeding-occasional suctioning required	23 (74.2)	11 (31.4)
Slight bleeding-frequent suctioning required	5 (16.1)	20 (57.1)
Moderate bleeding-frequent suctioning required	0 (0)	4 (11.4)
Severe bleeding	0 (0)	0 (0)

**Table 5:** Comparison of surgeon satisfaction profile among the patients during the treatment

Surgeon satisfaction profile	Group 1	Group 2
	n (%)	n (%)
Fully satisfied	4 (12.9)	0 (0)
Satisfied	22 (71)	10 (28.6)
Just satisfied	5 (16.1)	25 (71.4)
Not satisfied	0 (0)	0 (0)

Our study is being suggested to determine the role of the two different doses (1mcg/kg and 2mcg/kg), which have predominant sympathoadrenal suppression effects that are lowering BP and HR in facilitating controlled hypotension during FESS (primary objective). Our study shows that preinduction fentanyl can aid controlled hypotension by establishing a favorable hemodynamic state right after the induction of general anesthesia, allowing for the more effective use of commonly used hypotensive medications without developing acute tolerance and side effects.

## Discussion

There is a tonne of evidence that employing specific hypotensive medications during FESS can effectively achieve and maintain hypotensive anesthesia. However, there is currently only some limited source on Remifentanil research on the use of opioids during FESS hypotensive anaesthesia. While there is little research on usage of pre-induction fentanyl and infusion, fentanyl is frequently used and given soon before the induction of anesthesia. The research has not yet looked into how pre-induction fentanyl administration affects the maintenance and onset of controlled hypotension during FESS.

By lowering systemic vascular resistance (SVR) and cardiac output, controlled hypotension can be induced [1]. Any purposeful hypotension approach used during GA (general anesthesia) aims to reduce MAP (mean arterial pressure) in healthy individuals to levels between 50 and 65 millimetres of mercury, considerably lessening blood loss. There are pharmacological therapies for controlled hypotension which can be used both alone and in concert with other forms of care to minimize dosage requirements and, consequently, SE (side effects).

To help with controlled hypotension inhalation anesthetics like sevoflurane, isoflurane, vasodilators like sodium nitroprusside and NTG, trimetaphan camsilate, alprostadil (prostaglandin E1), alpha 2 adrenergic agonists such as Dexmedetomidine, clonidine, magnesium sulfate (MgS04), and short-acting opioids, propofol for total intravenous anesthesia (TIVA) is growing in acceptance [2].

In comparison with this study, other studies used different agents for controlled hypotension for FESS surgery. Dutta A, Choudhary P, et al. completed a 24-month experiment with 120 people. Their outcomes of fentanyl appear to be more effective in promoting regulated hypotension during FESS with regard to quantifiable hemodynamic points, surgeon satisfaction, and agreeable surgical conditions [1]. Jacobi K and Rickauer AJ, in 1999, 62 patients participated in a trial on preoperative Flupirtine during ambulatory FESS. They were randomized into two groups at random; Group F received preoperative F flupirtine (100 mg), while Group C received a placebo capsule administered orally 60 minutes before the start of anesthesia. They discovered that using flupirtine as a premedication improved the analgesia and hemodynamics of the perioperative period, resulting in reduced nasal bleeding and higher surgeon satisfaction ratings [6]. Elsharnouby NM et al. 2006; researched the use of MgSO4 as a hypotensive anesthetic method in FESS. The surgical time was shorter, but the anesthetic period was 10 minutes longer, delaying the anesthetic emergence, according to the results. The amount of blood lost, the amount of anesthesia needed, and the MAP and the HR were all dramatically lowered [7]. Richa F et al., in 2008, compared Dexmedetomidine and Remifentanil in a trial to reduce hypotension during tympanoplasty [8].

With the management of adequate doses of pre-induction fentanyl and infusion of fentanyl, it is possible to substitute the unreasonable practice of controlled hypotension by deepening anesthesia depth by increasing either inhalational concentration or intravenous dose, which would delay patients' emergence from anesthesia and recuperation in the post-op care room with packed nostrils, in addition to having adverse cardiovascular effects.

Strengths

Fentanyl gives suitable sedation and analgesia, reducing the use of inhalational agents. The side effects of inhalational agents are reduced with the use of fentanyl.

Limitations

The sample size could have been more and further studies with reduced doses of fentanyl may help attain the same outcomes with reduced side effects. 

## Conclusions

We concluded that regarding quantifiable hemodynamic endpoints, favorable operating conditions, surgeon satisfaction, and hypotensive agents in 68 patients who took part in the study, pre-induction fentanyl 2mcg/kg dosage appears better than 1mcg/kg dosage in permitting regulated hypotension during functional endoscopic sinus surgery. Pre-induction fentanyl with infusion can effectively control hypotension during FESS.

## References

[1] Choudhary P, Dutta A, Sethi N, Sood J, Rai D, Gupta M (2019). Pre-induction fentanyl dose-finding study for controlled hypotension during functional endoscopic sinus surgery. Indian J Anaesth.

[2] Boonmak P, Boonmak S, Laopaiboon M (2016). Deliberate hypotension with propofol under anaesthesia for functional endoscopic sinus surgery (FESS). Cochrane Database Syst Rev.

[3] Liu TC, Lai HC, Lu CH, Huang YS, Hung NK, Cherng CH, Wu ZF (2018). Analysis of anesthesia-controlled operating room time after propofol-based total intravenous anesthesia compared with desflurane anesthesia in functional endoscopic sinus surgery. Medicine (Baltimore).

[4] Jiwanmall M, Joselyn AS, Kandasamy S (2017). Intravenous clonidine as a part of balanced anaesthesia for controlled hypotension in functional endoscopic sinus surgery: A randomised controled trial. Indian J Anaesth.

[5] Escamilla Y, Cardesín A, Samara L (2019). Randomized clinical trial to compare the efficacy to improve the quality of surgical field of hypotensive anesthesia with clonidine or dexmedetomidine during functional endoscopic sinus surgery. Eur Arch Otorhinolaryngol.

[6] Jacobi K, Rickauer AJ (1999). [Prophylactic analgesia in functional endoscopic sinus surgery. Hemodynamics, surgical conditions, stress response]. Anasthesiol Intensivmed Notfallmed Schmerzther.

[7] Elsharnouby NM, Elsharnouby MM (2006). Magnesium sulphate as a technique of hypotensive anaesthesia. Br J Anaesth.

[8] Richa F, Yazigi A, Sleilaty G, Yazbeck P (2008). Comparison between dexmedetomidine and remifentanil for controlled hypotension during tympanoplasty. Eur J Anaesthesiol.

[9] Khalifa OS, Awad OG (2015). A comparative study of dexmedetomidine, magnesium sulphate, or glyceryltrinitrate in deliberate hypotension during functional endoscopic sinus surgery. Ain-Shams J Anaesthesiol.

[10] Nowak S, Ołdak A, Kluzik A, Drobnik L (2016). Impact of controlled induced hypotension on cognitive functions of patients undergoing functional endoscopic sinus surgery. Med Sci Monit.

[11] Bharathwaj DK, Kamath SS Comparison of dexmedetomidine versus propfol-based anesthesia for controlled hypotension in functional endoscopic sinus surgery.

[12] Chhabra A, Saini P, Sharma K, Chaudhary N, Singh A, Gupta S (2020). Controlled hypotension for FESS: A randomised double-blinded comparison of magnesium sulphate and dexmedetomidine. Indian J Anaesth.

[13] Shams T, El Bahnasawe NS, Abu-Samra M, El-Masry R (2013). Induced hypotension for functional endoscopic sinus surgery: A comparative study of dexmedetomidine versus esmolol. Saudi J Anaesth.

[14] Sahu BP, Nayak LK, Mohapatra PS, Mishra K (2021). Induced hypotension in functional endoscopic sinus surgery. A comparative study of dexmedetomidine and esmolol. Cureus.

[15] DeConde AS, Thompson CF, Wu EC, Suh JD (2013). Systematic review and meta-analysis of total intravenous anesthesia and endoscopic sinus surgery. Int Forum Allergy Rhinol.

